# P02.42. Non-touch biofield therapy: a systematic review of human randomized controlled trials reporting use of only non-physical contact treatment

**DOI:** 10.1186/1472-6882-12-S1-P98

**Published:** 2012-06-12

**Authors:** R Hammerschlag, B Marx, M Yamamoto, M Aickin

**Affiliations:** 1 Oregon College of Oriental Medicine, Portland, USA; 2 National College of Natural Medicine, Portland, USA; 3 Family & Community Medicine, University of Arizona, Tucson, USA

## Purpose

Biofield therapies, e.g. External Qigong, Healing Touch, Johrei, Reiki and Therapeutic Touch, commonly combine physical touch and non-physical contact to stimulate healing. Interpretation of such healing as mediated via electromagnetic and/or other types of biofield(s) is confounded by findings that light touch can lessen stress and reduce pain. The present review of human RCTs that report using only non-physical contact healing aims to develop evaluative criteria that reflect both the 'early-phase' (pilot study) nature of most biofield therapy trials and the real-world practice of these therapies.

## Methods

Biofield therapy RCTs were identified through June 2011 from databases, reference lists of systematic reviews and bibliographies web-posted by biofield therapy organizations. Included articles were peer-reviewed, English language prospective RCTs with at least one arm of verum therapy delivered with no physical contact and at least one arm of mimic therapy or active comparison treatment. Data extraction items were based on those of Astin et al 2000 to facilitate tracking of research progress in this field.

## Results

Of 74 identified RCTs, the 23 that met inclusion criteria examined 18 different conditions using 5-480 total minutes of treatment; 19 trials (83%) reported at least one outcome that favored verum treatment. Four trials reported at least one outcome that failed to reach statistical significance but produced a positive ‘separation test’ (Aickin 2007), indicating that further research is justified.

## Conclusion

Heterogeneity in treatment parameters across the included trials suggests lack of consensus on how to test biofield therapies within the RCT design. Variability of reporting of biofield therapy RCTs, in particular the frequent lack of unequivocal statements as to use of only hands-off procedures, hindered drawing robust conclusions. Nevertheless, the combined findings from statistical significance testing and separation testing indicate that further research under transparent conditions is justified to inform studies of mechanism(s) mediating non-physical contact healing.

